# miR-326 Targets Antiapoptotic Bcl-xL and Mediates Apoptosis in Human Platelets

**DOI:** 10.1371/journal.pone.0122784

**Published:** 2015-04-13

**Authors:** Shifang Yu, Huicong Huang, Gang Deng, Zuoting Xie, Yincai Ye, Ruide Guo, Xuejiao Cai, Junying Hong, Dingliang Qian, Xiangjing Zhou, Zhihua Tao, Bile Chen, Qiang Li

**Affiliations:** 1 The Department of Transfusion Medicine, The Second Affiliated Hospital of Zhejiang University School of Medicine, Hangzhou, China; 2 The Department of Transfusion Medicine, The First Affiliated Hospital of the Wenzhou Medical University, Wenzhou, China; 3 School of Basic Medical Sciences, Wenzhou Medical University, Wenzhou, China; 4 The Ningbo Central Blood Station, Ningbo, China; 5 The Department of Laboratory Medicine, The Third Affiliated Hospital of the Wenzhou Medical University, Ruian, China; Innsbruck Medical University, AUSTRIA

## Abstract

Platelets play crucial roles in hemostasis, thrombosis, wound healing, inflammation, angiogenesis, and tumor metastases. Because they are anucleated blood cells, platelets lack nuclear DNA, but they do contain mitochondrial DNA, which plays a key role in regulating apoptosis. Recent evidence has suggested that miRNAs are also involved in regulating gene expression and apoptosis in platelets. Our previous study showed that the expression of miR-326 increased visibly when apheresis platelets were stored *in vitro*. The antiapoptotic Bcl-2 family regulator Bcl-xL has been identified as a putative target of miR-326. In the present study, dual reporter luciferase assays were used to characterize the function of miR-326 in the regulation of the apoptosis of platelet cells. These assays demonstrated that miR-326 bound to the 3′-translated region of Bcl-xL. To directly assess the functional effects of miR-326 expression, levels of Bcl-xL and the apoptotic status of stored apheresis platelets were measured after transfection of miR-326 mimic or inhibitor. Results indicated that miR-326 inhibited Bcl-xL expression and induced apoptosis in stored platelets. Additionally, miR-326 inhibited Bcl-2 protein expression and enhanced Bak expression, possibly through an indirect mechanism, though there was no effect on the expression of Bax. The effect of miR-326 appeared to be limited to apoptosis, with no significant effect on platelet activation. These results provide new insight into the molecular mechanisms affecting differential platelet gene regulation, which may increase understanding of the role of platelet apoptosis in multiple diseases.

## Introduction

Platelets, small anucleated cells derived from bone marrow megakaryocyte precursors, make essential contributions to hemostasis, thrombosis, and other functions [1]. However, upon *in vitro* storage, platelets undergo two fundamental processes that determine their quality and life span: apoptosis and activation [2–5]. Apoptosis, programmed cell death, is regulated through two main pathways, intrinsic (or mitochondrial) and extrinsic. Studies show that the intrinsic pathway regulates the lifespan of platelets [3,4,6]. Because they are anucleated blood cells, platelets lack nuclear DNA. However, they contain mitochondrial DNA and associated mRNAs, including those encoding Bcl-2 family proteins. The Bcl-2 family is composed of anti-apoptotic (Bcl-2, Bcl-xL, and Mcl-1) and pro-apoptotic (Bax and Bak) members [7,8]. These play key roles in the intrinsic apoptosis pathway. Zhang et al. demonstrated that Bcl-xL played an important role in the regulation of platelet survival [4]. Bertino et al. observed degradation of Bcl-xL during platelet storage at 37°C [9].

Because platelets lack a functional nucleus, the expression of mRNAs such as Bcl-xL cannot be regulated by DNA transcription or replication. Consequently, the regulatory mechanism of Bcl-xL mRNA in platelet apoptosis is likely to be post-transcriptional. Up to 32% of all human genes are present in platelets at the mRNA level [10,11]. They undergo signal-dependent translational regulation [12–14]. Recent investigations have confirmed that platelets contain an abundance of many different kinds of miRNAs [15–17]. These are key regulators of mRNA translation. For this reason, the possible role of miRNAs in regulating mRNA translation in platelets has been studied and discussed [13,16,18]. Landry et al. observed a complex regulatory network in platelets that centers upon miRNAs [16]. Kannan et al. reported that platelets used miRNAs as translational regulators and played a crucial role in platelet apoptosis during storage [19].

A preliminary study demonstrated that the expression of miR-326 increased visibly when the apheresis platelets were stored *in vitro* [20]. Bioinformatic analysis indicated a putative target site within the 3′-translated region (UTR) of Bcl-xL. Mimics and inhibitors were used to further examine the potential relevance and mechanism of miR-326 in regulating platelet apoptosis. These modulated the expression of miR-326 in stored apheresis platelets. Results demonstrate that miR-326 inhibits the expression of Bcl-xL and induces the apoptosis of platelets.

## Materials and Methods

### Ethics Statement

Apheresis platelets were collected from healthy blood donors (7 males and 6 females). This and all other procedures were approved by the Human Ethics Committee of the First Affiliated Hospital of the Wenzhou Medical University and the Second Affiliated Hospital of Zhejiang University School of Medicine. All donors provided written informed consent.

### Preparation of leukocyte-depleted apheresis platelets (LDPs)

Apheresis platelets were collected from 13 healthy blood donors from the Wenzhou Blood Center and Ningbo Blood Center, Zhejiang, China (7 men and 6 women, 20–30 years old). The donors provided written informed consent, and the study was performed in accordance with the approval of an ethical committee. LDPs were prepared and used as previously reported [20]. To deplete white blood cells (WBCs), reticulocytes, and red blood cells (RBCs), the platelets were treated with anti-CD45^+^, anti-CD71^+^, and anti-CD235^+^ immunomagnetic beads according to the manufacturer’s recommendations (Pan Leukocyte; Invitrogen, Carlsbad, CA, U.S.). After treatment, 100,000 cells were analyzed using flow cytometry. The platelet count was approximately 2.0±0.5×10^11^/L, and no WBCs, RBCs, or reticulocytes were detected.

### Dual-luciferase reporter assay

Bioinformatic analysis was used to explore target genes and the main functions of miR-326. Results indicated a putative miR-326 target site within the 3′-UTR of Bcl-xL (gene: BCL2L1) and Bak (gene: BAK1). A fragment corresponding to the putative target site of miR-326 was ligated to the luciferase gene within psiCHECK-2 vector (Promega, Madison, WI, U.S.). As a control, a mutant version of the vector was prepared by modifying the seed sequence and confirmed the mutation by sequencing. 293T cells were seeded in a 48-well plate and transfected using lipofectamine 2000 (lipo 2000, Invitrogen, Carlsbad, CA, U.S.). Cells were harvested 48 h after transfection, and luciferase activity was measured using a dual-luciferase reporter assay system (Promega).

### Modulation of miR-326 expression

To investigate the role of miR-326 in the expression of the Bcl-xL mRNA and platelet apoptosis, LDPs were transfected with miR-326 miRNA agomir (mimic) or miRNA antagomir (inhibitor) (RiboBio Co., Ltd., Guangzhou, China). The negative controls, miRNA agomir control (miR-NC) and miRNA antagomir control (inhibitor-NC) (RiboBio Co., Ltd., Guangzhou, China) were structurally similar to the miR-326 mimic and inhibitor but were not predicted to target the 3′-UTR of Bcl-xL. Untransfected LDPs were used as blank control. Mimic, inhibitor, and negative controls were transiently transfected into LDPs at a final concentration of 50, 100, or 200 nM. The platelets were cultured under standard blood banking conditions and harvested at 24, 48, or 72 h.

### Transfection of LDPs with Bcl-xL and negative control siRNAs

To investigate the role of Bcl-xL mRNA in the expression of Bcl-xL protein and platelet apoptosis, we transfected LDPs with siBcl-xL, which was synthesized using the following sequences: siBcl-xL (Forward: 5′-CAGGGACAGCATATCAGAG-3′; reverse: 5′-GTCCCTGTCGTATAGTCTC-3′). A scrambled negative control (siNC, Ambion Inc., Austin, TX, U.S.) was also transfected in parallel. 5S rRNA was used as an endogenous control to normalize for differences in loading between samples. LDPs (2×10^8^/mL) were transfected using lipo 2000 at a final concentration of 50 nM and harvested at 48 h for assessment of Bcl-xL protein expression and apoptosis status.

### Analysis of platelet apoptosis

To assess the influence of miR-326 on platelet apoptosis, LDPs were harvested 24 h or 72 h after transfection with mimic or inhibitor. Flow cytometry (Annexin V, JC-1) and caspase-3 activity assay were used to assess the apoptosis status of platelets.

Mitochondrial depolarization is considered an early sign of apoptosis [6]. To assess Δ*Ψm* depolarization using flow cytometry, a commercially available JC-1 assay was used (BD Biosciences, San Jose, CA, U.S.). A total of 1–5×10^6^ LDPs were stained with JC-1 working fluid at room temperature for 30 min in the dark. Fluorescence intensity was analyzed on the Cytomics FC 500 (Beckman Coulter, Fullerton, CA, U.S.). Data on 50,000 platelets per sample were collected and analyzed. Each experiment was performed at least 3 times.

Annexin V is a Ca^2+^-dependent phospholipid-binding protein with high affinity for phosphatidylserine, a marker for both apoptosis and activation [21,22]. For annexin V staining, 1–5×10^6^ LDPs were resuspended in modified Tyrode’s buffer (without calcium or magnesium). Annexin V binding buffer was mixed with the LDPs and annexin V-FITC (BD Biosciences) at a ratio of 50:10:1. Samples were incubated at room temperature for 30 min in darkness and analyzed on the Cytomics FC 500 (Beckman Coulter).

A caspase-3 activation assay was performed based on the cleavage of the substrate DEVD-AFC (AFC: 7-amino-4-trifluoromethyl coumarin). A total of 1–5×10^6^ platelets were resuspended in 50 μl chilled cell lysis buffer. The platelets were incubated on ice for 10 min, and then 50 μl of 2× reaction buffer (containing 10 mM DTT) was added to each sample. An additional 5 μl of the 1 mM DEVD-AFC substrate (50 μM final concentration) was added, and the cells were incubated at 37°C for 1–2 h. The samples were read in 96-well plates in a fluorometer (SpectraMax M2, Molecular Devices Corporation, CA, USA) equipped with a 400-nm excitation filter and a 505 nm emission filter.

### Analyses of mRNA expression

Expression levels of mRNA were determined using One Step qRT-PCR. The RT-PCR Kit (TaKaRa Biotechnology (Dalian) Co., Ltd, Dalian, China) was used according to the manufacturer’s instructions. The primers were synthesized using the sequences listed in Table 1. Results were normalized to the expression of 5s rRNA. PCR cycling conditions were as follows: 50°C for 2 min, 95°C for 10 min, and 40 cycles of 95°C for 15 s and 60°C for 60 s. The qRT-PCR data were normalized using the 2-ΔΔ^Ct^ method. Melting curve analysis was performed to test the specificity and quality of the qRT-PCR amplification products. The results were calculated using StepOne Software v2.2.2 (Applied Biosystems, Foster City, CA, U.S.).

**Table 1:** Primers for qRT-PCR.

**Gene**	**Forward Primer (5'to3')**	**Reverse Primer (5'to3')**
5srRNA	TACGGCCATACCACCCTGAA	TAACCAGGCCCGACCCTGCT
Bcl-xL	TTACCTGAATGACCACCTA	ATTTCCGACTGAAGAGTGA
Bak	TGAGTACTTCACCAAGATTCA	AGTCAGGCCATGCTGGTAGAC
Mcl-1	AATCCCTGGAGCTCATCCTCCG	AGATGAGCGTGACAACTCGGCC
Bcl-2	TCGCCCTGTGGATGACTGA	CAGAGACAGCCAGGAGAAATA
Bax	GAGCGGCGGTGATGGA	TGGATGAAACCCTGAAGCAAA

### Western blotting

A total of 2.5×10^8^ LDPs were resuspended in 200 μl lysis buffer and incubated for 10 min at 4°C. The samples were centrifuged at 13,400 *g* for 30 s at 4°C, and the supernatant was collected. The proteins were separated by 10% sodium dodecyl sulfate polyacrylamide gel electrophoresis and then electroblotted onto PVDF membranes (Bio-Rad, Hercules, CA, U.S.). Membranes were blocked and incubated with polyclonal antibodies against Bcl-xL, Bcl-2, Mcl-1, Bax, Bak, or β-actin (Abcam, Cambridge, MA, U.S.). Then the membranes were incubated with horseradish peroxidase-conjugated IgG (Abgene Inc., Epsom, UK) and detected with Super Signal West Pico Chemiluminescent Substrate (Pierce, Rockford, IL, U.S.). Integrated density values were calculated using Quantity One (Bio-Rad).

### Platelet activation assays

Platelet apoptosis and activation are often considered related processes. For this reason, the effects of miR-326 on platelet activation were investigated simultaneously. The activation of platelets was assessed by measuring the following parameters: platelet surface activated GPIIb-IIIa expression (PAC-1 monoclonal antibody (mAb), which binds to activated GPIIb/IIIa complexes that are not occupied with adhesive proteins; BD Biosciences), exposure of *ɑ*-granule membrane proteins (CD62P anti-P-selectin mAb; BD Biosciences), and expression of lysosomal granule membrane protein (CD63; BD Biosciences). After transfection, 1–5×10^6^ LDPs were resuspended in modified Tyrode’s buffer and incubated with FITC-anti-CD62p,-CD63, or-PAC-1 at room temperature for 30 min in the dark. The intensity of fluorescence was analyzed using Cytomics FC 500 (Beckman Coulter). Data on 50,000 platelets per sample were collected and analyzed. Each experiment was performed at least 3 times.

### Statistical analysis

Data from at least 3 independent experiments are expressed as mean ± standard deviation (SD). Statistical analyses were performed using GraphPad prism 6 software (GraphPad Software, San Diego, CA, U.S.). Data showing comparisons between two groups were assessed using the Student’s t-test. Due to non-Gaussian distribution patterns of the results, non-parametric Kruskal-Wallis ANOVA was used to compare the data of groups. Statistical calculations were executed using SPSS software (v.14.0) (Chicago, IL, U.S.). *P*<0.05 was considered statistically significant.

## Results

### miR-326 interacts directly with the 3′-UTR of Bcl-xL

**Fig 1:**
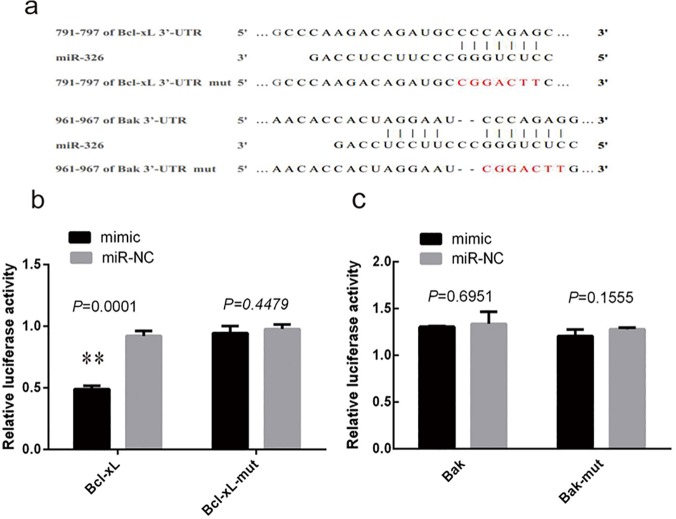
MiR-326 targets the 3′-untranslated region (UTR) of Bcl-xL but not that of Bak. (**a**) Putative miR-326 binding sequences in the 3′-UTR of the Bcl-xL and Bak genes and the mut sequences. (**b**) Transfection of miR-326 mimic compared to the negative control (miR-NC) significantly inhibited luciferase activity from a reporter vector containing the 3′-UTR of Bcl-xL (**P<0.01). However, there was no inhibition of a reporter vector with mutations in the miR-326-binding site (Bcl-xL-mut) (*P*>0.05). (**c**) Transfection of miR-326 did not significantly reduce the luciferase activity from reporter vectors containing the 3′-UTR of Bak or Bak-mut (*P*>0.05). Results represent the means ± SD of luciferase values in 3 independent experiments and are normalized to 1.0 for the miR-NC samples.

In a previous study, results showed that miR-326 was upregulated in platelets during storage, suggesting an association between miR-326 expression and apoptosis [20]. To identify potential targets of miR-326 that could be associated with apoptosis in platelets, we analyzed the 3′-UTR of mitochondrial Bcl-2 family genes. Putative miR-326 binding sites were identified at positions 791–797 (CCCAGAG) in the 3′-UTR of Bcl-xL and 961–967 (CCCAGAG) in the 3′-UTR of Bak (Fig 1A). To confirm the direct interaction of miR-326 with Bcl-xL and Bak, luciferase reporter plasmids were constructed by inserting wild-type or mutant fragments from the 3′-UTR regions of Bcl-xL or Bak into the vector psiCHECK-2. MiR-326 mimic and negative control miRNA (miR-NC) were cotransfected with these reporter plasmids into 293T cells, and luciferase activity was measured. MiR-326 was found to be significantly more effective than miR-NC at inhibiting the activity of the reporter vector containing the 3′-UTR of Bcl-xL (*P*<0.01). However, miR-326 did not inhibit the activity of the reporter vector containing the mutated 3′-UTR sequence, demonstrating that its effects were specific (Fig 1B). In contrast, miR-326 did not significantly inhibit the luciferase activity of the reporter vector containing either the wild type or mutated Bak 3′-UTR sequence (*P*>0.05) (Fig 1C). These results suggest that miR-326 directly targets the 3′-UTR of Bcl-xL, but not the 3′-UTR of Bak.

### MiR-326 negatively regulates the expression of Bcl-xL

**Fig 2:**
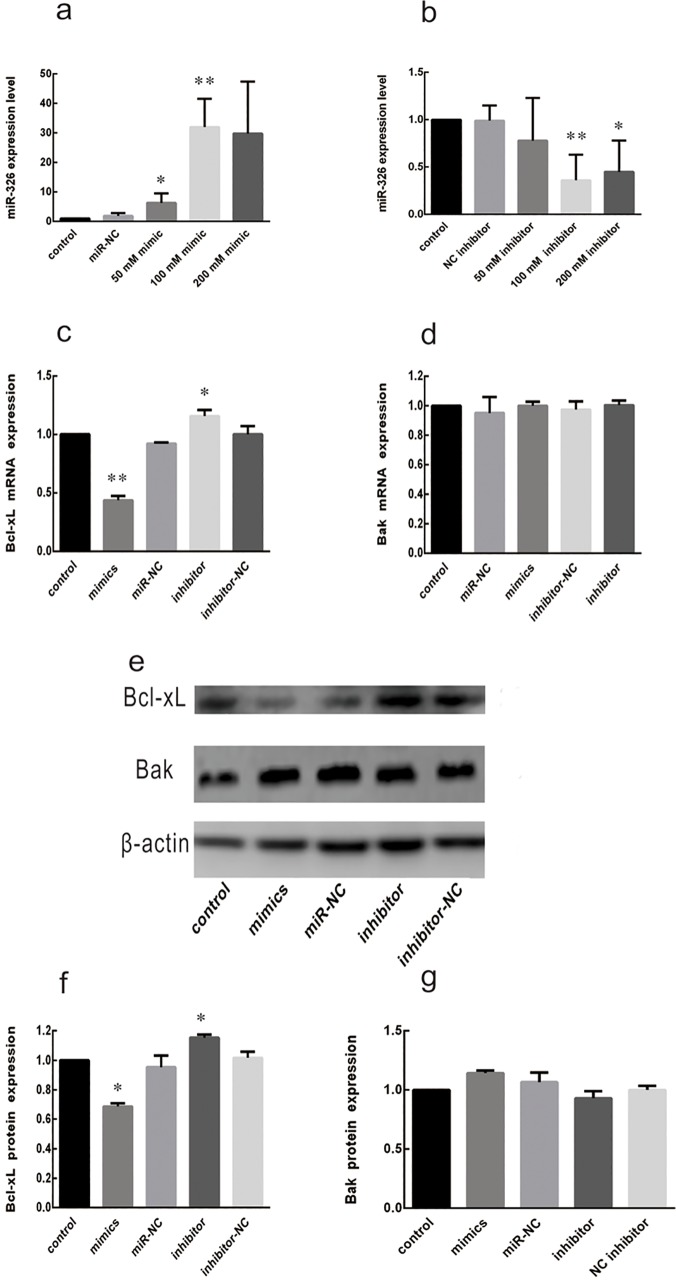
MiR-326 regulates the expression of endogenous Bcl-xL. (**a, b**) Transfection efficiency of miR-326 mimic and miR-326 inhibitor in leukocyte-depleted apheresis platelets (LDPs) were determined by qRT-PCR. LDPs were transfected using a transfection kit and mimic or inhibitor at a final concentration of 50, 100, or 200 nM and were collected after 24 h for analysis. A blank control sample and non-targeting negative RNA (miR-NC or inhibitor NC) were used as controls. *P<0.05, **P<0.01. (**c**) qRT-PCR of Bcl-xL mRNA was performed 24 h after transfection of LDPs as indicated. *P<0.05. (**d**) qRT-PCR of Bak mRNA was performed 24 h after transfection of LDPs as indicated. (**e–g**) Levels of Bcl-xL and Bak protein were determined by western blotting. The mean ± SD expression levels are shown. *P<0.05. Results are representative of 3 independent experiments.

To directly determine whether miR-326 modulates Bcl-xL expression, miR-326 mimic, miR-326 inhibitor, and the corresponding negative controls (miR-NC or inhibitor-NC) were transfected into LDPs at final concentrations of 50, 100, and 200 nM. As expected, miR-326 mimic specifically increased the expression of miR-326 24 h after transfection, and the miR-326 inhibitor specifically decreased miR-326 expression (Fig 2A and 2B). The effect on miR-326 expression for both the mimic and the inhibitor peaked at a concentration of 100 nM (26-fold increase by miR-326 mimic and 0.33-fold decrease by miR-326 inhibitor, *P*<0.01), and 50 nM, 100nM miR-326 mimic and 100 nM miR-326 inhibitor regulated Bcl-xL levels significantly (*P*<0.05, relative to NC). Considering the lower dose of 50 nM mimic were closer to physiological expression in stored platelets. Consequently, a final concentration of 50 nM mimic and 100 nM inhibitor was used in subsequent experiments.

To determine whether miR-326 mimic and miR-326 inhibitor also modulate Bcl-xL expression, platelets were collected 24h or 48 h after transfection and the Bcl-xL mRNA and protein levels were assessed by qRT-PCR and western analyses. As shown in Fig 2C, transfection of platelets with 50 nM miR-326 mimic decreased Bcl-xL mRNA levels (*P*<0.05, relative to miR-NC). However, after transfection, 100nM miR-326 inhibitor increased the Bcl-xL mRNA levels significantly (*P*<0.05, compared with inhibitor-NC) (Fig 2C). The Bak mRNA levels in platelets were not significantly influenced by the mimic or inhibitor (Fig 2D), which verified the specificity of the results. Consistent with the mRNA results, 48 h after transfection, the levels of Bcl-xL protein but not of Bak were lower in the presence of miR-326 mimic than in the presence of miR-NC (Fig 2E–2G). These results suggest that miR-326 can directly regulate the expression of Bcl-xL mRNA and protein in platelets *in vitro*.

### Transfection of LDPs with Bcl-xL siRNA promotes apoptosis

**Fig 3:**
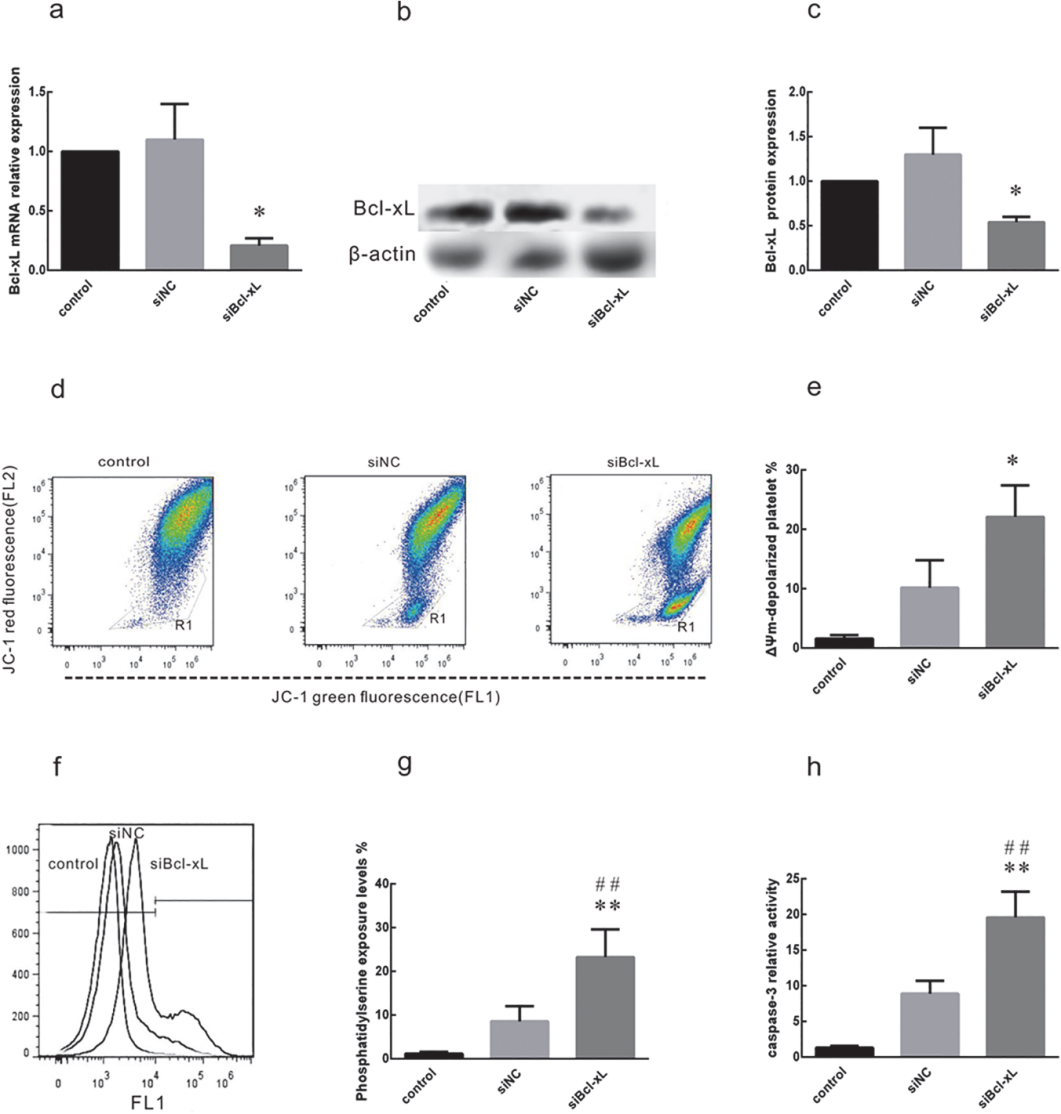
Bcl-xL siRNA effectively knocks down Bcl-xL mRNA and protein and induces platelet apoptosis. Leukocyte-depleted apheresis platelets (LDPs) were transfected with siBcl-xL or negative control siRNA (siNC) at a final concentration of 50 nM. LDPs without transfection were used as blank controls (control). Bcl-xL expression and apoptosis were assessed 48 h after transfection. (**a**) Expression levels of the Bcl-xL mRNA were assessed by qRT-PCR. 5s rRNA was used as an endogenous control. *P<0.05. (**b,c**) Bcl-xL protein levels were assessed by western blotting. β-actin was used as a loading control. *P<0.05. (**d, e**) Depolarization of platelet Δ*Ψm* was determined by JC-1 fluorochrome staining and flow cytometry. Histograms of platelets with JC-1 staining are shown for mock treated platelets (control) and platelets transfected with siNC or siBcl-xL. Depolarization is characterized as the decrease in the content of JC-1 aggregates, as reflected in the decrease of red (FL2) fluorescence. Dots inside R1 indicate that platelets are out of the main population and are considered to be undergoing Δ*Ψm* depolarization. *P<0.05. (**f, g)** Annexin V-FITC staining (R2) demonstrated that the annexin V positive cells in LDPs significantly increased as compared to the mock-transfected control and siNC (**P<0.01 vs. control, ^##^ *P*<0.01 vs. siNC). (**h**) The caspase-3 activity in LDPs was significantly increased as compared to control and negative siRNA (**P<0.01 vs. control, ^##^ *P*<0.01 vs. siNC). Values are expressed as the mean ± SD; n = 3 in each group.

To investigate the role of Bcl-xL in platelet apoptosis, siRNAs directed against Bcl-xL (siBcl-xL) and a negative control non-targeting siRNA (siNC) were synthesized. Efficient knockdown by siBcl-xL was verified at 48 h after transfection by qRT-PCR (Fig 3A) and western blotting (Fig 3B and 3C). siNC had no significant effect on Bcl-xL mRNA or protein expression.

To assess the effects of Bcl-xL knockdown on apoptosis in LPDs, flow cytometry (JC-1, Annexin V) and caspase-3 activity assays were performed 48 h after transfection. Mitochondrial depolarization (Δ*ψ*_*m*_) is considered to be an early sign of apoptosis [6]. The *ΔΨm* (as determined by JC-1 assay) was more pronounced in platelets transfected with siNC than in control mock-transfected platelets. However, the effect was greatest for platelets transfected with siBcl-xL (Fig 3D and 3E). Annexin V-FITC staining (Fig 3F and 3G) and caspase-3 activity levels (Fig 3H) were also significantly more pronounced for platelets transfected with siBcl-xL than for those transfected with siNC. These results indicate that Bcl-xL knockdown promotes apoptosis of platelets.

### MiR-326 promotes apoptosis in platelets

**Fig 4:**
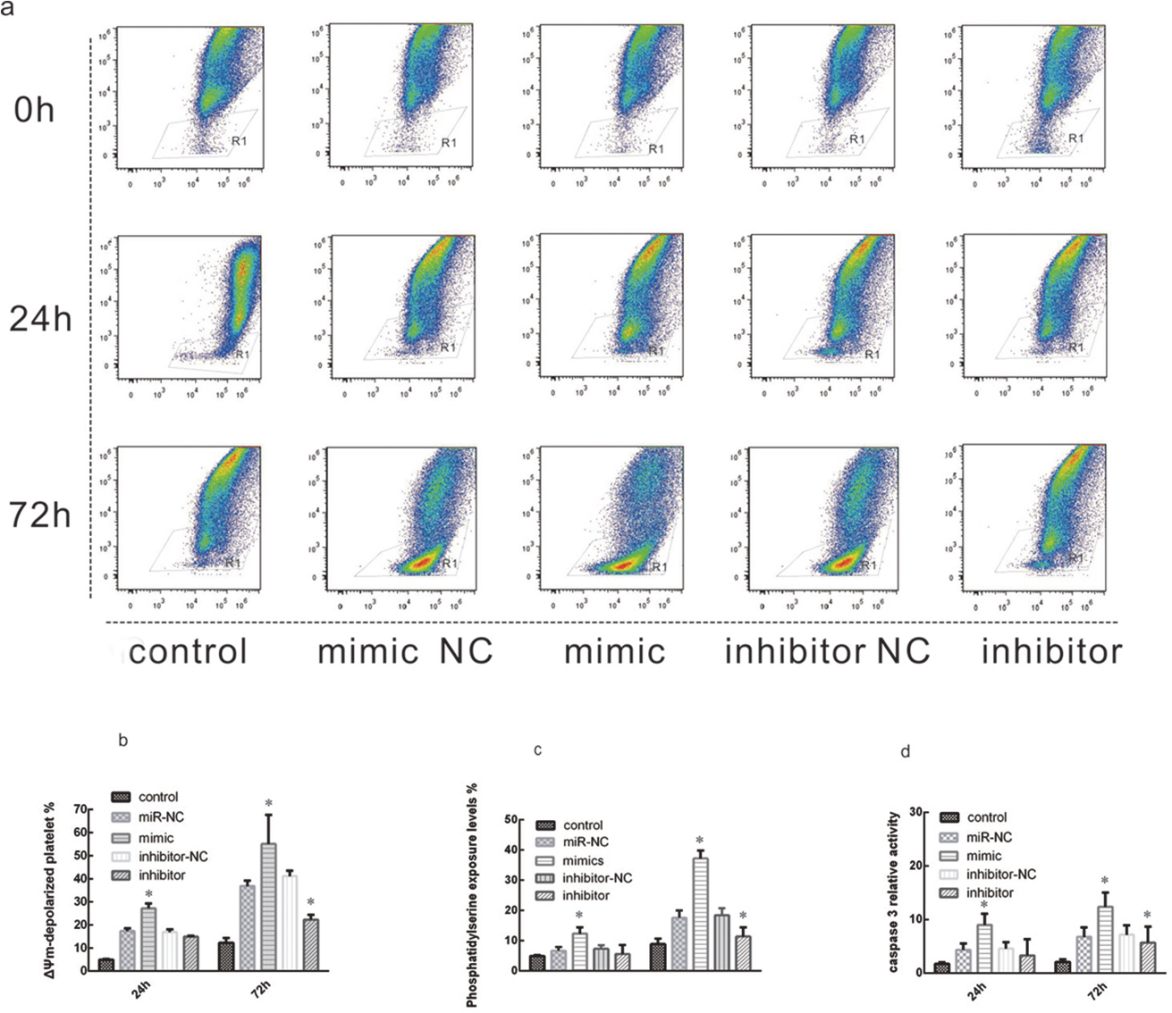
MiR-326 promotes platelet apoptosis. Leukocyte-depleted apheresis platelets (LDPs) were untransfected (control) or transfected with 50 nM of miR-326 mimic or a non-targeting negative control miRNA (miR-NC). LDPs were cultured under standard blood banking conditions and harvested at 24 or 72 h. Flow cytometry was used to analyze the apoptosis status of platelets after transfection. (**a, b**) Depolarization of *ΔΨm* was determined by JC-1 fluorochrome assay. *P<0.05. (**c**) Annexin V positive staining of platelets increased by nearly two-fold after transfection of miR-326 mimic. *P<0.05. (**d**) Overexpression of miR-326 mimic increased caspase-3 activity in LDP. *P<0.05.

To investigate the role of miR-326 in the promotion of platelet apoptosis, miR-326 mimic and miR-NC were transfected into LPDs and the levels of apoptosis were assessed. As shown in Fig 4A and 4B, the levels of Δ*Ψm* depolarization increased over time of storage. At 72 h, only 12.3±2.1% of control non-transfected platelets and 36.83±2.36% of miR-NC-transfected platelets exhibited mitochondrial depolarization, whereas 55.17±12.5% of miR-326 mimic-transfected platelets exhibited mitochondrial depolarization. In contrast, 22.3 ± 2.1 of miR-326 inhibitor-transfected platelets exhibited mitochondrial depolarization. These results show that storage of LPDs causes increased in membrane disruption and also indicate that the overexpression of miR-326 disrupts the mitochondrial membrane potential, which is an indicator of apoptosis.

To confirm these findings, annexin V-FITC staining and caspase-3 activation assays were performed. As in the JC-1 assay, the levels of phosphatidylserine exposure (annexin V staining) (Fig 4C) and caspase-3 activation (Fig 4D) increased over time and were most prominent in platelets transfected with miR-326 mimic. These results confirm the role of miR-326 in promoting apoptosis in platelets.

### Expression of additional Bcl-2 family members

**Fig 5:**
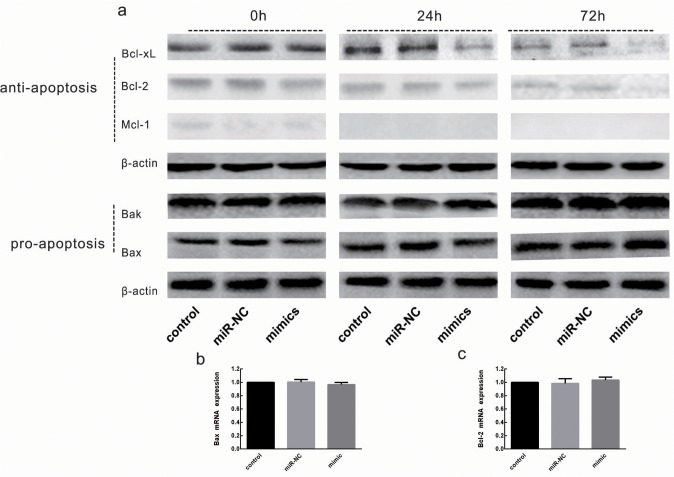
Expression of additional Bcl-2 family members after transfection. Lysates from washed platelets (20 μg) were subjected to western blot analysis for expression of proteins. (**a**) Western blotting analysis is shown for untransfected platelets (control) and platelets transfected with miR-326 mimic or non-targeting negative control (miR-NC). Leukocyte-depleted apheresis platelets (LDPs) were cultured under standard blood banking conditions and harvested at the indicated times after transfection. Results are representative of 3 independent experiments. (**b, c**) The effect of miR-326 mimic transfection on Bcl-2 and Bak mRNA expression (*P*>0.05 vs. siNC).

Bcl-xL is a member of a family of Bcl-2 proteins that are modulated during mitochondrial-dependent apoptosis, and modulation of these proteins serves as an additional marker of apoptosis. Studies have shown that Bcl-2 and Mcl-1 regulate Bak/Bax-dependent apoptosis [5,21]. To determine whether miR-326 modulates the levels of other Bcl-2 family members, the concentrations of Bcl-2 family members were examined in platelets over time and after transfection with miR-326 mimic or miR-NC. As shown in Fig 5A, after transfection, the levels of anti-apoptotic Bcl-2 family members changed. Levels of Bcl-xL and Bcl-2 decreased over time, and the decrease was more pronounced after transfection. Mcl-1 was detected only on day 0, and was not detected on the next days. However, the expression of the pro-apoptotic protein Bak showed mildly increased 72 h after transfection, whilelevels of the pro-apoptotic protein Bax did not change significantly. Bioinformatic analysis and luciferase assays suggested that miR-326 does not directly target the Bcl-2 or Bak promoters (data not shown). To confirm whether miR-326 can modulate the expression of Mcl-1, Bcl-2 or Bak, these mRNA were analyzed simultaneously. Results indicated that miR-326 had no significant effect on Bcl-2 and Bak mRNA expression, Mcl-1 mRNA was not detected (Fig 5B and 5C). For this reason, it is here speculated that the decreased expression is more likely an indication of an indirect response by which miR-326 triggers mitochondrial pathway-dependent platelet apoptosis. The increase of Bak might be attributable to the decrease in Bcl-xL levels, leading to a reduction in the Bcl-xL- mediated inhibition of Bak.

### Platelets activation by miR-326

**Fig 6:**
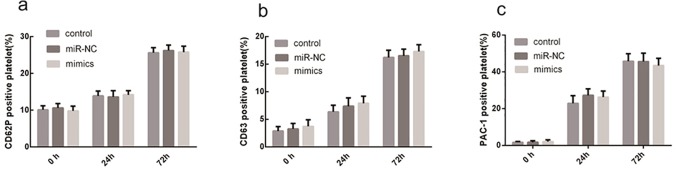
Platelets are not activated by miR-326 expression. Leukocyte-depleted apheresis platelets (LDPs) were untransfected (control) or treated with 50 nM miR-326 mimic or non-targeting negative control (miR-NC). LDPs were cultured under standard blood banking conditions and harvested at the indicated times. Expression and activity was determined by flow cytometry. (**a**) Relative number of P-selectin (CD62p)-positive platelets. (**b**) Relative number of CD63-positive platelets. (**c**) Relative number of PAC-1-positive platelets. Data were quantified from 3 separate experiments and are shown as mean ± SD.

Platelet apoptosis and activation are often considered related processes. Annexin V staining assays suggest that miR-326 promotes phosphatidylserine exposure of platelets, which is a marker of both apoptosis and activation [22,23]. To determine whether miR-326 induces platelet activation at the same time as the induction of apoptosis, CD62p, CD63, and PAC1 levels were measured using flow cytometry. The relative number of platelets positive for CD62p (Fig 6A), CD63 (Fig 6B), and PAC-1 (Fig 6C) was found to increase over time. However, there was no significant difference between miR-326 mimic- and miR-NC-transfected platelets. These results suggest that platelets are not activated by miR-326.

## Discussion

Mitochondria are generally considered critical in the life and death of cells. In addition to supplying energy, they have been demonstrated to be involved in the execution of apoptosis [24]. The mitochondria of human blood platelets work efficiently as energy factories, play crucial roles in the mitochondrial pathway of platelet apoptosis, and regulate the life span of platelets. Critical steps in the mitochondrial pathway involve the loss of mitochondrial membrane potential and the release of cytochrome *c* into the cytosol, where it triggers the activation of caspase-9. Because they are anucleated blood cells, platelets rely upon Bcl-2 family proteins, which consists of anti-apoptotic (e.g. Bcl-2, Bcl-xL and Mcl-1) and pro-apoptotic (e.g. Bax and Bak) members. These regulate the mitochondria-mediated intrinsic apoptotic pathway [22,23,25]. Pro-apoptotic proteins interact with the mitochondrial outer membrane, which leads to the release of cytochrome *c* and triggers the apoptotic cascade [3,26–28]. Anti-apoptotic proteins maintain cellular viability, possibly through the inhibition of Bax, Bak, or both [3,7,8,29,30]. Bcl-xL has been reported to be critically involved in the pathway of platelet apoptosis by restraining the pro-apoptotic proteins Bak and Bax [3].

MiRNAs are highly conserved non-protein-coding RNA molecules that are predicted to regulate approximately 60% of all human genes [31]. They play a significant role in regulation of gene expression at the post-transcriptional level [17,31]. Human platelets lack the nuclear components Drosha and DGCR8 but still have miRNA processing machinery, including Dicer, TAR RNA-binding protein 2, and Ago2. For this reason, they can still process pre-miRNA (which mostly originates from the cytoplasm of macrophages) into mature miRNA [16]. These miRNAs may be significant to the regulation of Bcl-xL and apoptosis in platelets. Results have shown that overexpression of miR-326 reduces both mRNA and protein levels of Bcl-xL. Inhibition of miR-326 expression was found to increase the expression of Bcl-xL mRNA and protein. The ability of miR-326 to regulate Bcl-xL expression is likely to take place through direct binding to the 3′-UTR region of Bcl-xL mRNA with complete complementarity to its seed region as shown by luciferase assay, but effects on other members of the Bcl-2 family are likely to be indirect. A significant increase in apoptosis activity was observed in platelets after miR-326 transfection. In this way, miR-326 is likely to promote apoptosis in platelets by attenuating Bcl-xL expression. Platelet apoptosis and activation are often considered related. However, evidence of the sequential dynamics and the magnitude of responses during platelet storage indicate that platelet activation and apoptosis are distinct phenomena involving different signaling pathways [32]. To determine whether miR-326 promotes platelet activation and to further explore the signaling pathways of platelet activation and apoptosis, we also investigated the effects of miR-326 on platelets. An increase in the expression of P-selectin, lysosomal granule membrane protein, and the activation of integrin ɑIIbβ3 in platelets was observed over time, but there was no significant difference for platelets transfected with miR-326 mimic versus miR-NC. These results suggest that platelets are not activated by miR-326.

Platelets, which are megakaryocyte-derived, anucleated blood cells, contribute to many different functions and disorders, including thrombocytosis, hemorrhage, atherosclerosis, tumor progression, and metastasis [33,34]. Like all lineages of blood cells, the steady state number of mature platelets is a result of a balance between their production and elimination. If this balance is disturbed, thrombocytopenia, or low platelet counts, can result. Thrombocytopenia is a common problem in the clinic, particularly for haematological and oncological practices because it can cause potentially fatal hemorrhagic episodes. Strategies that promote platelet survival by inhibiting apoptosis could be advantageous in some patients with thrombocytopenia. Bcl-xL constrains the pro-apoptotic activity of Bak to maintain the survival of platelets, and if Bcl-xL activity is compromised, the platelet life span and total number of platelets in circulation is reduced [3]. Data collected here indicate that miR-326 contributes to platelet apoptosis by inhibiting the expression of anti-apoptotic Bcl-xL, thus providing new insights into the molecular mechanisms affecting differential platelet gene regulation and improving understanding of the roles of platelets in multiple diseases.
